# Vestibular Migraine‐Like Syndrome in a Patient With Postttraumatic Headache and Medication‐Overuse Headache

**DOI:** 10.1155/crot/4950686

**Published:** 2026-02-04

**Authors:** Fumiyuki Goto, Takanobu Teramura, Shoji Kaneda, Kenji Okami, Koichiro Wasano

**Affiliations:** ^1^ Department of Otorhinolaryngology–Head and Neck Surgery, Tokai University School of Medicine, Isehara, Japan, u-tokai.ac.jp

**Keywords:** case report, medication-overuse headache, posttraumatic headache, vertigo, vestibular migraine

## Abstract

Vestibular migraine (VM) requires a migraine history according to ICHD‐3, but recurrent vertigo may also develop in secondary headache disorders. We report a 52‐year‐old man who experienced persistent headache and recurrent vertigo following a traffic accident. His headache fulfilled criteria for chronic posttraumatic headache and later medication‐overuse headache. Vertigo attacks lasted minutes to 1 h and were accompanied by photophobia, phonophobia, and motion sensitivity, resembling VM despite no migraine history. Preventive therapy with amitriptyline and valproate reduced vertigo frequency and analgesic overuse. This case suggests that VM‐like syndromes can occur with secondary headaches, where migraine‐oriented prophylaxis may be useful.

## 1. Introduction

Vestibular migraine (VM) is one of the most common causes of recurrent vertigo, but its diagnosis requires a history of migraine, as defined in the International Classification of Headache Disorders, 3rd edition (ICHD‐3) [1]. Posttraumatic headache (PTH) is a frequent sequela of head injury and can evolve into chronic headache or medication‐overuse headache (MOH) [2, 3]. In some patients, vertigo resembling VM occurs during the course of PTH or MOH, presenting diagnostic and therapeutic challenges [4, 5].

## 2. Case Report

A 52‐year‐old man developed persistent headache and recurrent vertigo following a traffic accident. The headache began immediately after trauma and fulfilled ICHD‐3 criteria for chronic PTH [1]. He later used nonsteroidal anti‐inflammatory drugs almost daily, meeting criteria for MOH [3].

The vertigo was characterized by recurrent, spinning attacks lasting from several minutes to 1 h, accompanied by photophobia, phonophobia, and motion‐induced worsening, resembling VM [4]. No typical migraine headaches were reported. On referral to our clinic, his Dizziness Handicap Inventory score was 60, and the Hospital Anxiety and Depression Scale showed elevated scores (anxiety 16, depression 14). Posturography revealed increased sway (open eyes 140, closed eyes 208).

No progressive hearing loss or persistent imbalance was detected. Although peripheral vestibular injury could not be fully excluded due to lack of vestibular evoked myogenic potential (VEMP) and video head impulse test (vHIT), the episodic and reversible nature of vertigo, together with absence of auditory deterioration, argued against peripheral vestibulopathy.

A diagnosis of chronic PTH complicated by MOH was made. To evaluate treatment response, the frequency of vertigo attacks was quantified as the number of monthly vertigo episodes. Before treatment, he experienced about 10 episodes per month. After initiation of preventive therapy with amitriptyline (10 mg) and valproate (200 mg), vertigo frequency decreased to 4 episodes during the first month, 2 in the second month, and only 1 in the third month.

## 3. Discussion

This case highlights a diagnostic dilemma where vertigo closely resembled VM but occurred in a patient without migraine history, in the context of PTH and MOH. According to ICHD‐3, such a case cannot be classified as VM [1]. Therefore, despite the strong clinical resemblance, this presentation cannot be diagnosed as VM and must instead be regarded as a “VM‐like syndrome.” We explicitly added this clarification to avoid any misunderstanding regarding VM diagnostic boundaries.

The relationship between PTH, MOH, and vertigo may reflect overlapping mechanisms involving central sensitization and migraine‐related pathways [2, 3]. Excessive analgesic use likely aggravated both headache and vertigo, consistent with the pathophysiology of MOH [3].

Differential diagnoses such as perilymph fistula, traumatic endolymphatic hydrops, or semicircular canal dehiscence were considered but appeared less likely given the transient course, lack of auditory progression, and favorable response to migraine prophylaxis. Similar diagnostic challenges in distinguishing vestibular symptoms associated with migraine from secondary causes have been emphasized in previous reviews [4, 5].

Importantly, despite not fulfilling VM criteria, the patient responded to migraine‐oriented preventive treatment. This suggests that therapeutic strategies for VM [4, 5] may be applicable to patients with VM‐like syndromes arising in secondary headache contexts. Clinicians should recognize such cases and consider preventive therapy for both vertigo and headache control.

## 4. Conclusion

VM‐like syndromes may develop in patients with PTH and MOH, even in the absence of migraine history. While strict diagnostic criteria prevent labeling these cases as VM [1], migraine‐oriented prophylaxis can be clinically effective. The interaction between PTH, MOH, and vertigo likely reflects overlapping mechanisms involving central sensitization, dysregulated trigeminovascular activation, and impaired vestibulo‐thalamo‐cortical modulation. Analgesic overuse may further amplify these pathways, worsening both headache and vestibular symptoms. The notable reduction in monthly vertigo episodes after prophylactic therapy supports the concept that migraine‐related neural circuits were involved, even though the underlying headache disorder remained secondary.

Similar cases have been described in the literature, where trauma‐induced migraine‐like headaches or vestibular symptoms respond to migraine‐oriented therapy despite not fulfilling VM criteria. These findings underscore that strict adherence to VM diagnostic criteria is necessary, but therapeutic approaches may still draw from migraine management when the clinical phenotype suggests migraine‐related mechanisms. Awareness of such presentations may help optimize treatment and reduce analgesic overuse in posttraumatic patients.

## Funding

This work was supported by a Grant‐in‐Aid for Scientific Research (KAKENHI), Grant Number 25K12820, from the Japan Society for the Promotion of Science (JSPS).

## Consent

Written informed consent was obtained from the patient for publication of this case report.

## Conflicts of Interest

The authors declare no conflicts of interest.

## Data Availability

Data supporting the findings of this study are available from the corresponding author upon reasonable request.
